# Ptomaine Poisoning.—I

**Published:** 1911-08-19

**Authors:** H. J. Hutchens

**Affiliations:** Heath Professor of Comparative Pathology and Bacteriology in the University of Durham.


### Hospital Clinics.

### PTOMAINE POISONING.—I.

By H. J. HUTCHENS, D.S.O., D.P.H. Oxon., M.A.
Heath Professor of Comparative Pathology and Bacteriology in the University of Durham.

Ptomaine poisoning is a popular rather than a scientific term used to describe an illness following, and attributable to, the consumption of a particular article of food. In nearly all cases the symptoms are of the nature of a gastro-enteritis, and it is further characteristic of the disease that by far the greater number of those who have partaken of the incriminated food suffer from the disease. The term ptomaine poisoning is inexact, because it leads by inference to the assumption that the symptoms are due to ptomaines, while in fact, as will be shown, these substances are not the cause of the disease. The word ptomaine (πτῶμα, a corpse) was introduced by the Italian toxicologist Selmi to describe certain chemical substances more or less allied to the vegetable alkaloids which had been found in putrescent meat and decomposing albuminous matter.

### Bacteria and Putrefaction.

In the earlier part of the last century, as the outcome of investigations by Panum and others, it had been shown that substances of a poisonous nature, chemically resembling the vegetable alkaloids, were present in decomposing animal matter, and these substances were later made the subject of an elaborate investigation by Selmi, and later still by Brieger and others. The result was that a number of definite chemical compounds, having many properties in common with one another, were isolated from decomposing flesh, etc. When the relation of bacteria to putrefaction had been proved by Pasteur it was recognised that these alkaloidal bodies were the products of bacterial activity. And the further researches of Pasteur and Koch, which established the micro-organic nature of the infective diseases of man and the lower animals, gave a great stimulus to the investigation of the products of micro-organisms, because it very soon became evident that the infecting micro-organisms exerted their effects by means of chemical products elaborated by them in the medium in which they happened to be growing, whether that medium were the living body or an artificial culture medium. The truth of this view of the nature of the action of micro-organisms was proved in 1880, when Pasteur showed that by the inoculation of filtered, and therefore sterilised, cultures of the organism of fowl cholera he was able to reproduce the disease in susceptible animals just as though he had inoculated the living organisms.

### Varieties of Ptomaines.

The detection of alkalodial bodies in unsound meat led to the opinion that the symptoms following the consumption of such meat was directly due to them. This explains the origin of the expression ptomaine poisoning. As the result of the work of Selmi, Brieger, and later observers, a considerable knowledge of ptomaines has been acquired, and a large number have been isolated and their chemical composition determined. Vaughan gives a list of fifty-seven different ptomaines, the majority of which have received names indicating more or less, in most cases, the sources whence they were originally obtained. At first the ptomaines were regarded as characteristic products of the decomposition of animal matter, and the word itself would indicate that this was their origin. The majority of ptomaines have no doubt been obtained from putrescent meat or flesh, but they are also found under other conditions. Muscarin, for instance, is the active principle of poisonous mushrooms, but has also been obtained from decomposing flesh.

### What is a Ptomaine?

A ptomaine may be defined as "an organic chemical compound, basic in character, and formed by the action of bacteria on nitrogenous matter" (Vaughan). Ptomaines "are to be regarded as extracellular products of bacterial activity. They do not originate within the bacterial cell, and therefore are not to be looked upon as direct metabolic products of the cell protoplasm, but rather as secondary cleavage products." Many of the ptomaines belong to the benzene series and are derivatives of pyridine; others are hydro-carbon derivatives. Most of them, either in their chemical or physiological reactions resemble the vegetable alkaloids. "Some may be obtained from a variety of decomposing substances, irrespective of the type of organisms present." They cannot, therefore, be regarded as the specific products of bacteria. They are found in only very small amounts in decomposing animal matter, and it is only when meat is in so advanced a stage of decomposition as to be totally unfit for human food that they are present at all. Moreover, many of the ptomaines are non-poisonous, and the majority of those that act as poisons exert their influence on the nervous system rather than on the alimentary system, which is further evidence against their being the cause of the symptoms of food-poisoning, since in the great majority of cases the latter are of the nature of a gastro-enteritis. (There is, of course, a definite class of epidemics of food-poisoning, in which the active substance is of the nature of a neuro-muscular poison. This, however, is not a ptomaine, and reference will again be made to this particular form of food-poisoning under the head of Botulism.)

### Food Poisoning.

On various grounds, therefore, ptomaines can no longer be considered to be the cause of food-poisoning. It would perhaps be exceeding the limits of what has been definitely established experimentally to say that ptomaines never cause symptoms of food-poisoning, but all the available evidence is against their playing any part in the production of those symptoms. The ptomaines have, therefore, largely lost their interest for the bacteriologist. But to the toxicologist they are of supreme importance, to which fact the recollection of a recent criminal trial will bear witness.

Food-poisoning is now attributed to the specific poisons of bacteria. Chemical investigation has shown the presence in culture media in which bacteria have been grown of numerous substances of a highly complex nature, ptomaines, tox-albumins, albumoses, etc., but all attempts so far to determine the nature of the specific bacterial poisons by chemical means have failed. The specific products to which the action of bacteria on the tissues is due are known by the generic name *toxin*. Toxins differ from all such substances as ptomaines, tox-albumins, etc., in that they are specific. They are "the result of synthetical processes occurring within the bacterial cell." Bacterial toxins are the most poisonous substances known. It has been shown, for instance, that 1 c.c. of a particular broth culture of tetanus contained 0.025 gramme of organic matter, and that that quantity contained sufficient toxin to kill 100,000 mice. Even assuming that the whole of the 0.025 gramme of organic matter was toxin, which of course it was not, the example serves to show the extremely poisonous nature of bacterial toxins.

### Toxins of Food.

It is to these toxins that the symptoms following the invasion of the body by bacteria are to be attributed. And when it was shown that food-poisoning could not be regarded as a ptomaine effect it was thought that the symptoms must be due to bacterial toxins present in the meat. This is undoubtedly true in some cases, as will be shown later. But Gärtner's observations during an epidemic of food-poisoning at Frankenhausen in 1888 showed that food-poisoning was at least sometimes an infective disease. And, as a matter of fact, in most cases food-poisoning is a specific infective disease, and is the result of poisoning by bacterial toxins. The toxins may be present in the food before it is eaten, or they may be elaborated in the human body. The former condition gives rise to a toxæmia while the latter is a true infection, resulting from the ingestion of organisms contained in the food which has been eaten. The latter condition is due to a small group of organisms known as the "enteritidis" or Salmonella group, the members of which are bacteriologically closely related to the typhoid and colon bacilli. It is possible that occasionally they give rise to a toxæmia, but in the great majority of cases food-poisoning due to these organisms is an infective disease.

Food-poisoning is, therefore, the result of the action of the specific toxins of bacteria on persons who consume meat or other food infected with living organisms or their toxins, or both. The non-specific products should also, perhaps, be included; for though the evidence so far available is against the view that they take any part in the production of food-poisoning, it cannot be stated as a definitely ascertained fact that they never exert any influence. This definition at once excludes from the category of food-poisoning all cases of poisoning following the consumption of food containing arsenic, lead, strychnine, or other well-defined chemical substance, whether administered intentionally for criminal purposes or taken by accident. On the other hand, the generally accepted use of the term does not include such diseases as enteric fever, Malta fever, etc., though these are also the direct result of eating food specifically contaminated with the organisms of those diseases.

### Epidemics.

If epidemics of food-poisoning be classified according to the nature of the organism concerned, they may be divided conveniently into three groups:

1. Epidemics caused by organisms belonging to the enteritidis group;
2. Epidemics due to *Bacillus botulinus;*
3. Epidemics assciated with other organisms.

1. Epidemics caused by organisms belonging to the enteritidis group.

In the Frankenhausen epidemic Gärtner isolated from the spleen of the fatal case and also from the meat which had caused the epidemic an organism which had not hitherto been described, and to which he gave the name *B. enteritidis*. This observation entirely altered the views then held with regard to food-poisoning, in that it showed that in some cases, at least, the disease was a true infection. In many of its characters the *B. enteritidis* (Gärtner) resembles the typhoid and colon bacilli, but shows certain differences from those organisms, and notably in its effects upon the human body. Some ten years later (1898) Durham and then de Nobelé showed that from epidemics of food-poisoning two closely related organisms had been isolated. They found that while serum obtained from any of the affected persons in a given epidemic agglutinated the organism which was the cause of the epidemic, it did not always agglutinate the bacillus from another clinically identical epidemic. This showed, therefore, that epidemics of food-poisoning might be due to either of two organisms, and that these organisms could only be distinguished from one another by resort to agglutination tests. These organisms are known respectively as *B. enteritidis* (Gärtner), which was the organism originally found, and *B. enteritidis* (Aertrycke), representing the other type, so called by an epidemic at Aertrycke in Belgium, investigated by de Nobelé.

According to Sacquépée, 80 per cent. of epidemics of food-poisoning are due to these bacilli.

The enteritidis bacilli, like the other members of the typhoid-colon group to which they belong, are short cocco-bacilli, occasionally growing out into long filamentous forms, gram-negative, staining more deeply at the poles than in the centre, flagellated, motile, not liquefying gelatin and not forming spores. The group includes the various dysentery bacilli, the typhoid bacillus, the bacillus paratyphoid *a* (which causes a disease clinically indistinguishable from enteric fever) the bacilli of the enteritidis group and the numerous colon bacilli.

*(To be continued.)*

